# Interaction of acute heart failure and acute kidney injury on in-hospital mortality of critically ill patients with sepsis: A retrospective observational study

**DOI:** 10.1371/journal.pone.0282842

**Published:** 2023-03-08

**Authors:** Tianyang Hu, Wanjun Yao, Yu Li, Yanan Liu

**Affiliations:** 1 Precision Medicine Center, The Second Affiliated Hospital of Chongqing Medical University, Chongqing, China; 2 Department of Anesthesiology, Wuhan No.1 Hospital, 430030, Wuhan, Hubei, China; 3 Department of Nephrology, Chongqing Bishan District People’s Hospital (Bishan Hospital Affiliated to Chongqing Medical University), Chongqing, China; 4 Department of Nephrology, Rheumatology and Immunology, Jiulongpo District People’s Hospital, Chongqing, China; Mansoura University, EGYPT

## Abstract

**Background:**

The present study aimed to evaluate the synergistic impact of acute heart failure (AHF) and acute kidney injury (AKI) on in-hospital mortality in critically ill patients with sepsis.

**Methods:**

We undertook a retrospective, observational analysis using data acquired from the Medical Information Mart for Intensive Care-IV (MIMIC-IV) database and eICU Collaborative Research Database (eICU-CRD). The effects of AKI and AHF on in-hospital mortality were examined using a Cox proportional hazards model. Additive interactions were analyzed using the relative extra risk attributable to interaction.

**Results:**

A total of 33,184 patients were eventually included, comprising 20,626 patients in the training cohort collected from the MIMIC-IV database and 12,558 patients in the validation cohort extracted from the eICU-CRD database. After multivariate Cox analysis, the independent variables for in-hospital mortality included: AHF only (HR:1.20, 95% CI:1.02–1.41, P = 0.005), AKI only (HR:2.10, 95% CI:1.91–2.31, P < 0.001), and both AHF and AKI (HR:3.80, 95%CI:13.40–4.24, P < 0.001). The relative excess risk owing to interaction was 1.49 (95% CI:1.14–1.87), the attributable percentage due to interaction was 0.39 (95%CI:0.31–0.46), and the synergy index was 2.15 (95%CI:1.75–2.63), demonstrated AHF and AKI had a strong synergic impact on in-hospital mortality. And the findings in the validation cohort indicated identical conclusions to the training cohort.

**Conclusion:**

Our data demonstrated a synergistic relationship of AHF and AKI on in-hospital mortality in critically unwell patients with sepsis.

## Introduction

Sepsis is a life-threatening organ dysfunction produced by a dysregulated host response to infection and it is a continual worry in critically ill patients not only because of its high prevalence but also due to the high mortality [1–3]. Sepsis and septic shock are the major cause of death, morbidity, and cost overruns in the critically ill patient [4,5]. In recent years, significant attempts have been made to enhance the treatment of sepsis, nevertheless, critically ill patients may have a death rate as high as about half of all patients [6,7]. Such an exceptionally high mortality rate may be attributable to the advancement of various organ failures including heart failure, kidney failure, and respiratory failure [8].

Acute heart failure (AHF) is a frequent consequence in severely unwell individuals with sepsis. It is notable that sepsis in the context of AHF significantly affects results including greater fatality rates. Patients with severe sepsis/septic shock and concomitant AHF had a 75% death rate 1-year postdischarge [9]. Furthermore, sepsis-related acute kidney failure (AKI) is another prevalent consequence of critically ill patient and is linked with unacceptable morbidity and death [10]. It is characterized by rapid impairment of renal function accompanied with sepsis. AKI occurs in 40–50% of septic patients and increases mortality 6-fold [11]. Both AHF and AKI might enhance the risk of death in severely unwell sepsis patients. However, whether AHF and AKI collaborated to synergistically enhance the risk of in-hospital mortality in critically unwell sepsis patients remains mainly unknown. Hence, the present study intended to obtain data extracted from the Medical Information Mart for Intensive Care-IV (MIMIC-IV) database and eICU Collaborative Research Database (eICU-CRD), two large public databases to clarify the interaction effect of AHF and AKI on in-hospital mortality in critically ill patients with sepsis.

## Methods

### Data source

All data were extracted from the eICU-CRD [12] and the MIMIC-IV database [13]. After completing the web-based training classes and the Protecting Human Research Participants assessment, we got approval to harvest data from the eICU-CRD and MIMIC-IV databases. The study was ethically approved by an affiliated of the Massachusetts Institute of Technology (No.27653720). All patients-related information in the database is anonymous, so there is no need to obtain the informed consent of the patients. This study is described in conformity to the Strengthening the Reporting of Observational Studies in Epidemiology (STROBE) statement, and was managed to conform to the tenets of the Declarations of Helsinki.

### Cohort selection

**Fig 1:**
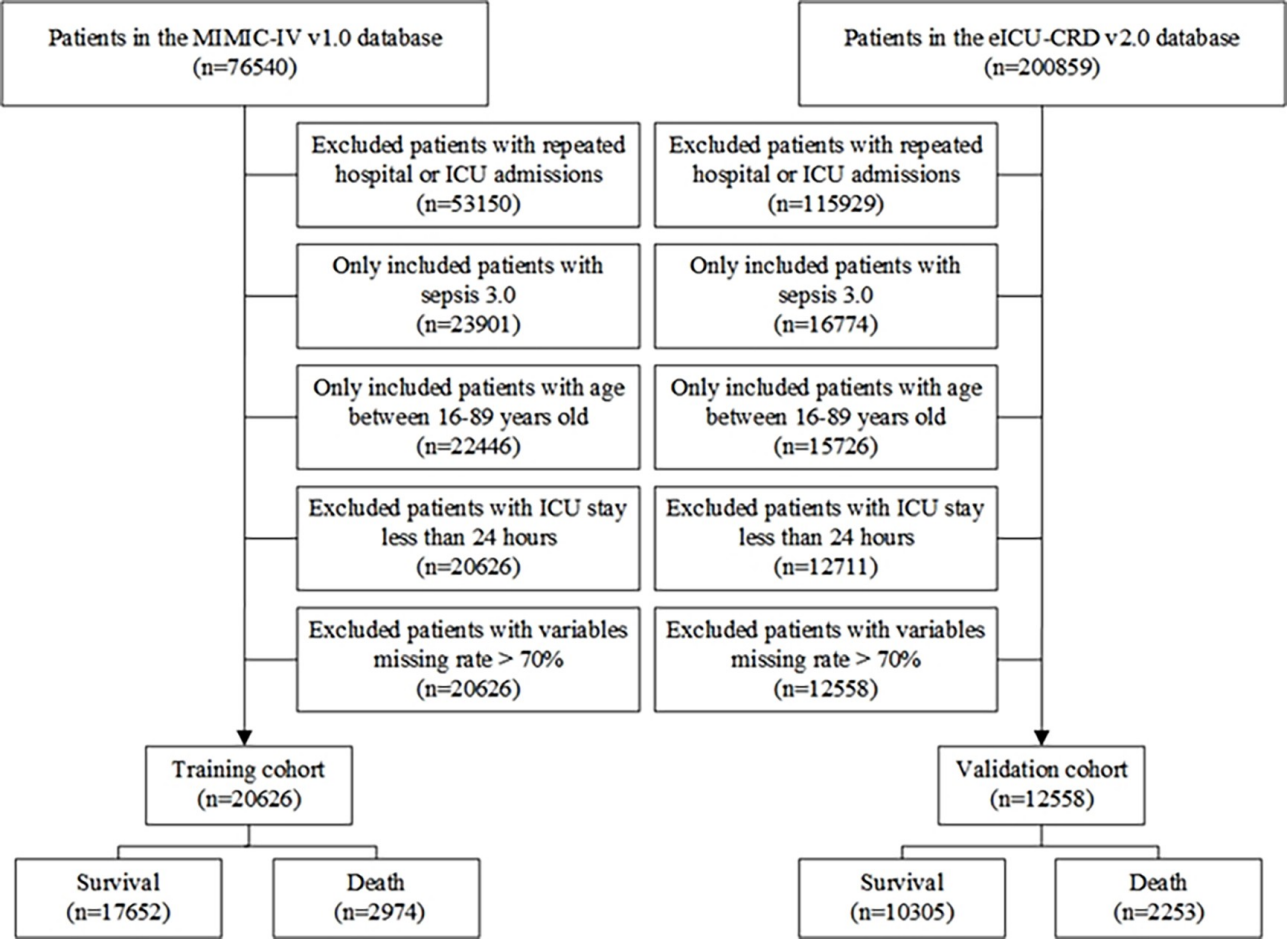
The flow chart of this study.

Patients diagnosed with sepsis based on the sepsis 3.0 criteria [14] over 16 years older at their first ICU admission were included in the present study. Patients with one of the following characteristics were excluded: 1) less than 16-year-old at initial admission to ICU; 2) patients with ICU stay shorter than 24 hours; 3) patients with repeated ICU hospitalizations; 4) patients with variables missing rate > 70% [15]. A total of 20,626 patients in the MIMIC-IV database allocated to the training cohort and 12,588 patients in the eICU database assigned to the validation cohort were eventually included in this investigation (Fig 1).

### Data collection and outcomes

Baseline characteristics and admission information including: age, gender, weight, ethnicity, comorbidities, drugs were collected. Moreover, severity scores including sequential organ failure assessment (SOFA) score, the oxford acute severity of illness score (OASIS), the acute physiology score III (APSIII) were also collected, the severity scores are useful tools to quantify the degree of organ dysfunction or failure present on ICU admission which has been widely used for prognosis prediction in ICU settings [16]. Vital signs and initial laboratory results, and intervention on the first day of their ICU admission were also recorded in this study. In addition, length of the hospital (LOS) was also included.

The primary outcome was in-hospital mortality.

### Definitions

The definition of AKI is according to KDIGO-AKI criteria [17]. And the diagnosis of AHF is based on the International Classification of Diseases (ICD) code.

### Statistical analysis

The Chi-square test or Fisher’s test was correctly done to examine the differences between groups. All participants were divided into four categories according to the complications of AHF and AKI (neither AKI nor AHF, AKI only, AHF only, both AKI and AHF) (neither AKI nor AHF, AKI only, AHF only, both AKI and AHF). All variables were originally estimated by univariate Cox regression analysis, and only statistically significant variables were integrated into the multivariable Cox regression model. The Kaplan-Meier analysis was performed to estimate overall survival, and the differences between the curves were examined using the log-rank test [18]. R package ‘interaction R’ was used to calculate the additive interaction between AKI and AHF based on three factors: the relative excess risk due to interaction (RERI), the attributable proportion due to interaction (AP), and the synergy index (SI) [19]. The 95%CI of RERI, AP, and SI were assessed using a method proposed by Zou GY [20].

We discovered that BNP values were not acquired for more than half of the patients in this research (89.1% in the training cohort and 70.3% in the validation cohort absent) and the BNP values were crucial for the diagnosis and prognosis of AHF patients. If we had utilized the BNP measurements as the covariate, we would have had a huge number of missing values. Instead, we employed the presence or absence of BNP values as the covariate. Thus, a flag indicating whether BNP was recorded was added as a covariate in our analysis.

The statistical analysis was performed using statistical software SPSS 23.0 or R software. P < 0.05 was considered statistically significant.

## Results

### Baseline characteristics

A total of 33,184 patients were eventually included in the current investigation, comprising 20,626 patients in the training cohort collected from the MIMIC-IV database and 12,558 patients in the validation cohort taken from the eICU-CRD database. The flow pattern of the included population was given in Fig 1. There were 17,652 survival and 2,974 death in the training cohort, 10,305 survival and 2,253 death in the validation cohort. Of the 20,626 critically ill sepsis patients in the training cohort, 5,187 (25.1%) patients had AKI, 1,706 (8.3%) patients had AHF, 1,452 (7.0%) patients had both AKI and AHF, 12,281 (59.6%) patients had neither AKI nor AHF. Of the 12,558 critically ill sepsis patients in the validation cohort, 2,964 (23.6%) individuals had AKI, 946 (7.5%) patients had AHF, and 504 (4.0%) patients had both AKI and AHF, 8,144 (64.9%) patients had neither AKI nor AHF. Tables 1 and 2 showed the baseline characteristics of all patients in the training cohort classified by AHF and AKI and S1 Table revealed the baseline characteristics of all patients in the validation cohort grouped by AHF and AKI. Those who complicated with AKI or AHF had greater rates of comorbidities, and risk factors compared to those complicated with AKI or AHF (P < 0.05, respectively, Tables 1, 2 and S1). The in-hospital mortality in the training cohort of the neither AKI nor AHF, AKI only, AHF only, both AKI and AHF were 7.0%, 23.5%, 11.5%, and 48.3%, respectively (Table 1). The in-hospital mortality in the validation cohort of the neither AKI nor AHF, AKI only, AHF only, both AKI and AHF were 9.2%, 31.0%, 19.3%, and 59.5%, respectively (S1 Table).

**Table 1:** The basic demographic and outcomes of all patients in training cohort categorized by AHF and AKI.

Characteristics	Neither AKI or AHF	AKI only	AHF only	Both AKI and AHF	P value
N	12281	5187	1706	1452	-
Age, years old	63.3 ± 15.9	64.8 ± 15.1	70.7 ± 13.3	70.8 ± 12.7	<0.001
Gender, male, n (%)	7260 (59.1)	3205 (61.8)	914 (53.6)	837 (57.6)	<0.001
Ethnicity, n (%)					0.006
White	8271 (67.3)	3367 (64.9)	1126 (66)	935 (64.4)	
Black	1314 (10.7)	647 (12.5)	198 (11.6)	184 (12.7)	
Others	2696 (22.0)	1173 (22.6)	382 (22.4)	333 (22.9)	
Body mass index, kg/m2	28.3 ± 8.2	29.3 ± 7.7	30.1 ± 9.2	29.9 ± 8.6	<0.001
Comorbidities, n (%)					
Hypertension	5055 (41.2)	2212 (42.6)	685 (40.2)	599 (41.3)	0.197
Diabetes	3653 (29.7)	1628 (31.4)	496 (29.1)	462 (31.8)	0.058
Congestive heart failure	3361 (27.4)	1389 (26.8)	466 (27.3)	383 (26.4)	0.773
Myocardial infarct	2095 (17.1)	837 (16.1)	275 (16.1)	277 (19.1)	0.046
Chronic kidney disease	2457 (20.0)	1058 (20.4)	368 (21.6)	270 (18.6)	0.197
Liver disease	1919 (15.6)	798 (15.4)	257 (15.1)	245 (16.9)	0.504
COPD	2852 (23.2)	1220 (23.5)	691 (40.5)	540 (37.2)	<0.001
Atrial fibrillation	3014 (24.5)	1648 (31.8)	800 (46.9)	739 (50.9)	<0.001
Valve disease	10323 (84.1)	4253 (82)	1168 (68.5)	933 (64.3)	<0.001
Cardiac arrhythmia	4566 (37.2)	2351 (45.3)	1066 (62.5)	960 (66.1)	<0.001
PVD	1169 (9.5)	795 (15.3)	232 (13.6)	275 (18.9)	<0.001
Hypothyroidism	1515 (12.3)	643 (12.4)	287 (16.8)	237 (16.3)	<0.001
Clinical outcomes					
LOS, days	7.8 (5.0, 13.7)	10.2(5.9, 18.7)	10.6(6.9, 16.1)	23.8 (7.8, 20.1)	<0.001
Hospital mortality, n (%)	855 (7.0)	1220 (23.5)	197 (11.5)	702 (48.3)	<0.001

AHF, acute heart failure, AKI, acute kidney injury, COPD, chronic obstructive pulmonary disease, PVD, peripheral vascular diseases, LOS, length of hospital.

**Table 2:** The baseline characteristics of all patients in training cohort categorized by AHF and AKI.

Characteristics	Neither AKI or AHF	AKI only	AHF only	Both AKI and AHF	P value
N	12281	5187	1706	1452	-
Charlson index, points	5.7 ± 2.0	5.7 ± 1.9	5.7 ± 1.9	5.8 ± 1.9	0.707
Interventions, n (%)					
MV use	7945 (64.7)	3969 (76.5)	1049 (61.5)	1003 (69.1)	<0.001
RRT use	126 (1.0)	687 (13.2)	47 (2.8)	195 (13.4)	<0.001
Vasopressors	5793 (47.2)	3477 (67)	913 (53.5)	944 (65.0)	<0.001
Drug usage, n (%)					
ACEI/ARB	3442 (28.0)	1402 (27.0)	480 (28.1)	407 (28.0)	0.576
β blockers	8165 (66.5)	3424 (66)	1139 (66.8)	952 (65.6)	0.827
CCB	2585 (21.0)	1080 (20.8)	342 (20.0)	302 (20.8)	0.815
Diuretic	8208 (66.8)	3457 (66.6)	1147 (67.2)	962 (66.3)	0.941
Severity scores, points					
SOFA	6.6 ± 2.8	6.5 ± 2.7	6.4 ± 2.7	6.6 ± 2.8	0.350
OASIS	34.9 ± 9.4	34.8 ± 9.1	34.7 ± 9.3	34.9 ± 9.5	0.838
APS III	55.1 ± 16.0	54.6 ± 15.7	54.7 ± 15.4	55.6 ± 16.4	0.504
GCS	11.9 ± 3.8	10.9 ± 4.4	12.1 ± 3.6	11.3 ± 4.1	<0.001
Vital signs					
Heart rate, bpm	89.2 ± 19.5	92.0 ± 21.3	90.6 ± 21.1	91.5 ± 21.2	<0.001
Respiratory rate, bpm	18.9 ± 5.9	19.7 ± 6.4	20.7 ± 6.3	20.9 ± 6.7	<0.001
MAP, mmHg	83.4 ± 18.0	80.9 ± 19.6	82.0 ± 18.7	80.6 ± 19.1	<0.001
Laboratory results					
WBC, × 10^9^/L	12.3 ± 4.4	12.8 ± 5.5	12.7 ± 4.6	12.6 ± 4.1	0.010
HGB, g/dL	11.1 ± 2.3	10.8 ± 2.4	11.0 ± 2.2	10.7 ± 2.4	<0.001
Platelets, × 10^9^/L	206.4 ± 87.2	193.5 ± 94.3	217.4 ± 81.0	214.6 ± 93.9	<0.001
Albumin, g/dL	3.1 ± 0.5	3.1 ± 0.6	3.2 ± 0.5	3.2 ± 0.6	<0.001
Bilirubin, mmol/L	1.5 ± 0.3	2.6 ± 0.9	1.1 ± 0.4	1.2 ± 0.4	<0.001
Aniongap, mEq/L	14.2 ± 3.9	16.1 ± 5.3	15.3 ± 4.1	16.6 ± 4.5	<0.001
Bicarbonate, mEq/L	23.3 ± 4.4	21.9 ± 5.1	24.0 ± 5.5	23.1 ± 5.6	<0.001
BUN, mg/dL	22.8 ± 8.8	32.1 ± 8.1	32.3 ± 9.6	39.7 ± 10.5	<0.001
Creatinine, mg/dL	1.1 ± 0.5	2.1 ± 0.7	1.4 ± 0.6	2.2 ± 1.0	<0.001
Lactate, mmol/L	2.7 ± 0.7	4.0 ± 1.3	2.6 ± 0.7	3.4 ± 1.1	<0.001
BNP (tested)	828 (6.7)	515 (9.9)	511 (30.0)	399 (27.5)	<0.001

MV, mechanical ventilation, RRT, renal replacement therapy, ACEI/ARB, Angiotensin converting enzyme inhibitors/Angiotensin receptor blockers, CCB, Calcium calcium blockers, SOFA, sequential organ failure assessment, OASIS, oxford acute severity of illness score, APSIII, acute physiology score III, GCS, lasgow coma scale, MAP, mean arterial pressure, WBC, white blood cell, BUN, blood urea nitrogen, BNP, brain natriuretic peptide.

### Independent variables related with in-hospital death in severely unwell sepsis patients

**Fig 2:**
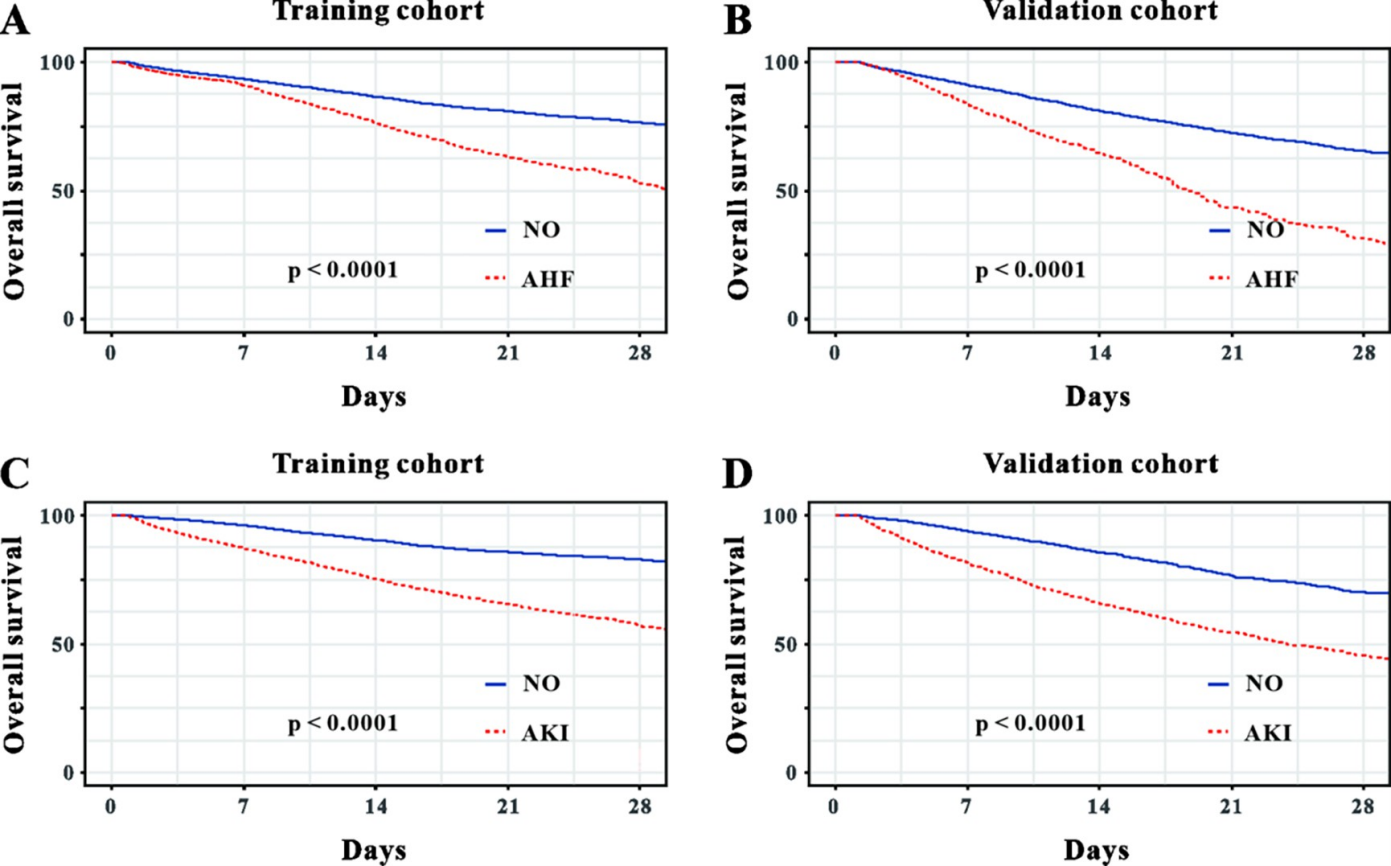
The Kaplan-Meier curves of overall survival for all patients stratified by acute heart failure (**A** and **B**) or acute kidney injury (**C** and **D**) in the training cohort and in the validation cohort.

**Fig 3:**
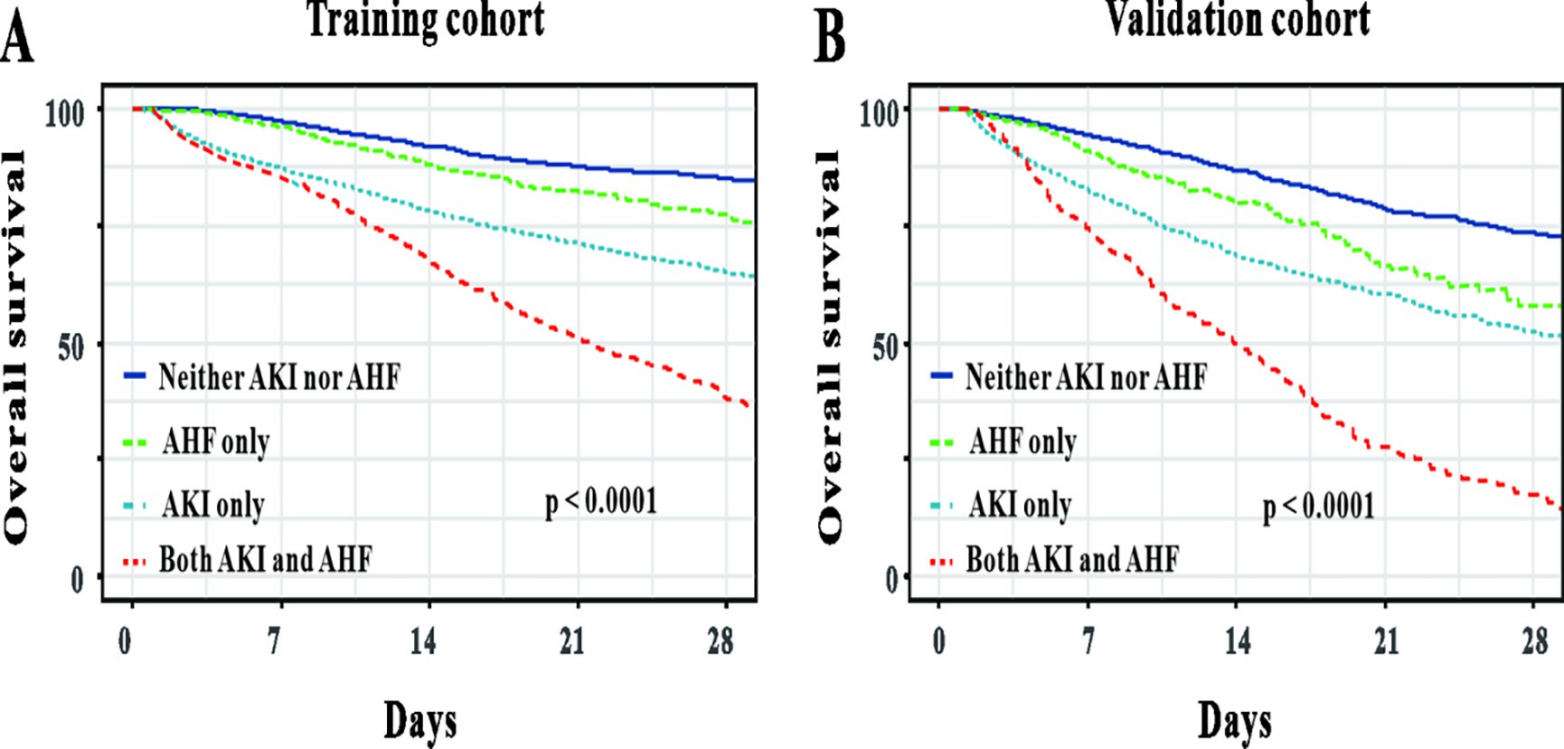
The Kaplan-Meier curves of overall survival for all patients stratified by the presence or absence of acute heart failure and acute kidney injury.

Cox regression was used to evaluate the important predictive factors of in-hospital mortality in critically ill patients with sepsis. Multivariate Cox analyses demonstrated that the complications of AKI and AHF had prognostic effect on sepsis patients in both of the cohorts (P < 0.05, Table 3 and S2 Table). And this was further confirmed by Kaplan-Meier analysis. Patients complicated with AKI or AHF had the worse overall survival rate compared with those not compicated with AKI or AHF both in the training cohort and in the validation cohort (Fig 2A–2D). In addition, Kaplan-Meier analysis revealed that those compicated with both AKI and AHF had the worst prognosis compared to those compicated with AKI only or AHF only or neither AKI nor AHF (Fig 3A and 3B).

**Table 3:** Cox analyses of factors associated with in-hospital mortality in training cohort.

	Univariate analysis		Multivariate analysis	
	HR (95%CI)	P value	HR (95%CI)	P value
Age	1.01 (1.00–1.02)	0.003	1.02 (1.01–1.02)	<0.001
Gender, male	0.95 (0.88–1.02)	0.141		
Ethnicity				
White	1.16 (0.95–1.27)	0.421		
Black	1.00 (0.87–1.14)	0.941		
Others	Ref.	-		
Body mass index	1.00 (0.99–1.00)	0.167		
Comorbidities				
Hypertension	1.06 (0.98–1.14)	0.114		
Diabetes	0.99 (0.92–1.07)	0.807		
Congestive heart failure	0.98 (0.90–1.06)	0.640		
Myocardial infarct	1.05 (0.95–1.15)	0.325		
Chronic kidney disease	0.95 (0.87–1.04)	0.284		
Liver disease	0.97 (0.88–1.07)	0.578		
COPD	1.00 (0.92–1.08)	0.939		
Atrial fibrillation	1.25 (1.16–1.35)	<0.001	1.03 (0.92–1.15)	0.629
Valve disease	1.53 (1.38–1.70)	<0.001	1.21 (1.09–1.34)	<0.001
Cardiac arrhythmia	1.32 (1.31–1.42)	<0.001	1.11 (0.99–1.24)	0.060
PVD	1.15 (1.04–1.28)	0.008	1.22 (1.10–1.36)	<0.001
Hypothyroidism	1.08 (0.97–1.20)	0.146		
Charlson index	1.00 (0.99–1.01)	0.858		
Interventions				
MV use	1.29 (1.18–1.40)	<0.001	1.02 (0.92–1.12)	0.770
RRT use	2.26 (2.05–2.49)	<0.001	1.02 (0.92–1.13)	0.704
Vasopressors	1.86 (1.72–2.01)	<0.001	1.36 (1.24–1.50)	<0.001
Drug usage				
ACEI/ARB	0.98 (0.90–1.06)	0.609		
β blockers	0.97 (0.90–1.05)	0.418		
CCB	1.01 (0.92–1.05)	0.418		
Diuretic	0.98 (0.91–1.06)	0.666		
Severity scores				
SOFA	1.19 (1.18–1.20)	<0.001	1.08 (1.07–1.09)	<0.001
OASIS	1.08 (1.07–1.09)	<0.001	1.05 (1.04–1.05)	<0.001
APS III	1.02 (1.01–1.03)	<0.001	1.01 (1.00–1.01)	<0.001
GCS	0.92 (0.91–0.93)	<0.001	0.94 (0.93–0.95)	<0.001
Vital signs				
Heart rate	1.00 (0.99–1.01)	0.759		
Respiratory rate	1.05 (1.04–1.06)	<0.001	1.02 (1.01–1.03)	<0.001
MAP	0.99 (0.99–1.01)	0.072		
Laboratory results				
WBC	1.00 (0.99–1.01)	0.069		
HGB	0.96 (0.94–1.03)	0.214		
Platelets	1.00 (0.99–1.02)	0.497		
Albumin	0.85 (0.81–0.90)	<0.001	0.86 (0.81–0.0.91)	<0.001
Bilirubin	1.00 (0.99–1.01)	0.818		
Aniongap	0.99 (0.98–1.00)	0.497		
Bicarbonate	1.00 (1.00–1.01)	0.135		
BUN	1.02 (1.01–1.03)	0.006	1.01 (1.01–1.02)	<0.001
Creatinine	1.03 (1.01–1.15)	0.008	0.99 (0.96–1.02)	0.486
Lactate	1.04 (1.03–1.05)	0.009	1.07 (1.06–1.08)	<0.001
BNP (tested)	0.66 (0.59–0.75)	<0.001	0.63 (0.56–0.71)	<0.001
Complications				
Neither AHF nor AKI	Ref.	-	Ref.	-
AHF only	1.38 (1.18–1.61)	<0.001	1.20 (1.02–1.41)	0.005
AKI only	2.62 (2.40–2.86)	<0.001	2.10 (1.91–2.31)	<0.001
Both AHF and AKI	4.85 (4.38–5.36)	<0.001	3.80 (3.40–4.24)	<0.001

HR, hazard ratio, 95%CI, 95% confidence index, COPD, chronic obstructive pulmonary disease, PVD, peripheral vascular diseases, MV, mechanical ventilation, RRT, renal replacement therapy, ACEI/ARB, Angiotensin converting enzyme inhibitors/Angiotensin receptor blockers, CCB, Calcium calcium blockers, SOFA, sequential organ failure assessment, OASIS, oxford acute severity of illness score, APSIII, acute physiology score III, GCS, glasgow coma scale, MAP, mean arterial pressure, WBC, white blood cell, BUN, blood urea nitrogen, BNP, brain natriuretic peptide, AHF, acute heart failure, AKI, acute kidney injury.

### Subgroup analysis

To further validate the interaction impact of AKI and AHF on in-hospital mortality, exploratory subgroup analyses were done in various subgroups. As exhibited in Table 4, subgroup analysis demonstrated that compared to patients who had neither not AHF nor AKI, patients with one complication (AHF or AKI) showed an increased risk of in-hospital mortality regardless of age, gender, congestive heart failure or not, with chronic kidney disease or not both in the training cohort and in the validation cohort (all P < 0.01, Table 4). And patients who had both two complications exhibited a largely elevated risk of in-hospital mortality compared to those patients who had one complication regardless of age, gender, congestive heart failure or not, chronic kidney disease or not both the training cohort and in the validation cohort (all P < 0.01, Table 4).

**Table 4:** Pre-specified subgroup analysis with hazards ratio of in-hospital mortality for patients with sepsis.

	Training cohort		Validation cohort	
	HR (95%CI)	P value	HR (95%CI)	P value
Age ≥ 65 years old				
Neither AHF nor AKI	Ref.	-	Ref.	-
AHF only	1.25 (1.06–1.48)	0.009	1.88 (1.58–2.24)	<0.001
AKI only	2.23 (2.01–2.47)	<0.001	2.59 (2.31–2.89)	<0.001
Both AHF and AKI	4.03 (3.60–4.51)	<0.001	5.00 (4.31–5.81)	<0.001
Age < 65 years old				
Neither AHF nor AKI	Ref.	-	Ref.	-
AHF only	1.09 (0.69–1.73)	0.707	2.06 (1.33–3.17)	0.001
AKI only	3.78 (3.20–4.47)	<0.001	2.57 (2.10–3.13)	<0.001
Both AHF and AKI	6.35 (5.04–7.99)	<0.001	5.83 (4.30–7.91)	<0.001
Male				
Neither AHF nor AKI	Ref.	-	Ref.	-
AHF only	1.46 (1.18–1.80)	<0.001	2.05 (1.64–2.55)	<0.001
AKI only	2.66 (2.37–2.99)	<0.001	2.60 (2.27–2.96)	<0.001
Both AHF and AKI	5.35 (4.69–6.11)	<0.001	5.67 (4.74–6.78)	<0.001
Female				
Neither AHF nor AKI	Ref.	-	Ref.	-
AHF only	1.28 (1.02–1.61)	0.033	2.10 (1.66–2.66)	<0.001
AKI only	2.59 (2.26–2.95)	<0.001	2.62 (2.27–3.02)	<0.001
Both AHF and AKI	4.18 (3.58–4.88)	<0.001	5.12 (4.17–6.29)	<0.001
With Congestive heart failure				
Neither AHF nor AKI	Ref.	-	Ref.	-
AHF only	1.56 (1.18–2.06)	0.002	2.00 (1.51–2.63)	<0.001
AKI only	2.61 (2.21–3.09)	<0.001	2.37 (1.83–3.08)	<0.001
Both AHF and AKI	4.77 (3.93–5.79)	<0.001	5.57 (4.33–7.17)	<0.001
Without congestive heart failure				
Neither AHF nor AKI	Ref.	-	Ref.	-
AHF only	1.31 (1.08–1.57)	0.005	1.87 (1.49–2.36)	<0.001
AKI only	2.62 (2.36–2.90)	<0.001	2.65 (2.38–2.94)	<0.001
Both AHF and AKI	4.87 (4.33–5.47)	<0.001	4.75 (3.97–5.69)	<0.001
With chronic kidney disease				
Neither AHF nor AKI	Ref.	-	Ref.	-
AHF only	1.56 (1.12–2.17)	0.008	1.56 (1.10–2.21)	0.013
AKI only	2.89 (2.36–3.53)	<0.001	1.58 (1.26–1.98)	<0.001
Both AHF and AKI	5.47 (4.33–6.91)	<0.001	3.67 (2.82–4.78)	<0.001
Without chronic kidney disease				
Neither AHF nor AKI	Ref.	-	Ref.	-
AHF only	1.34 (1.12–1.59)	0.001	2.13 (1.77–2.56)	<0.001
AKI only	2.56 (2.32–2.82)	<0.001	2.85 (2.56–3.17)	<0.001
Both AHF and AKI	4.70 (4.21–5.25)	<0.001	5.73 (4.89–6.73)	<0.001

HR, hazard ratio, 95%CI, 95% confidence index, AHF, acute heart failure, AKI, acute kidney injury.

### Additive interaction of AKI and AHF on in-hospital mortality in critically ill patients with sepsis

As indicated in Table 5, in the training cohort, the calculated RERI was 1.49 (95% CI:1.14–1.87), suggesting that there would be 1.49 relative excess risks attributable to the additive interaction between AHF and AKI. AP indicated that 39% of the overall chances of in-hospital death were due to the interaction of AHF and AKI. In addition, SI was 2.15 (95% CI, 1.75–2.63), demonstrating that the risk of in-hospital mortality in both AHF and AKI patients was 2.15 times as high as the sum of hazards in patients presenting just one single condition. Similar to the findings in the training cohort, in the validation cohort, the estimated RERI was 1.38 (95% CI:0.78–2.04), suggesting that there would be 1.38 relative excess risks attributable to the additive interaction between AHF and AKI. AP indicated that 30% of the overall chances of in-hospital death were due to the interaction of AHF and AKI. In addition, SI was 1.64 (95% CI:1.32–2.04), demonstrated that the risk of in-hospital mortality in both AHF and AKI patients was 1.64 times as high as the sum of hazards in patients presenting just one single condition.

**Table 5:** Measures for estimation of biological interaction between AHF and AKI for the risk of in-hospital mortality in patients with sepsis.

Biological interaction	Training cohort		Validation cohort	
	Estimate	95%CI	Estimate	95%CI
RERI	1.49	1.14–1.87	1.38	0.78–2.04
AP	0.39	0.31–0.46	0.30	0.18–0.40
SI	2.15	1.75–2.63	1.64	1.32–2.04

AHF, acute heart failure, AKI, acute kidney injury, RERI, relative excess risk due to interaction; AP, attributable proportion; SI, synergy index, CI, confidence interval.

**Fig 4:**
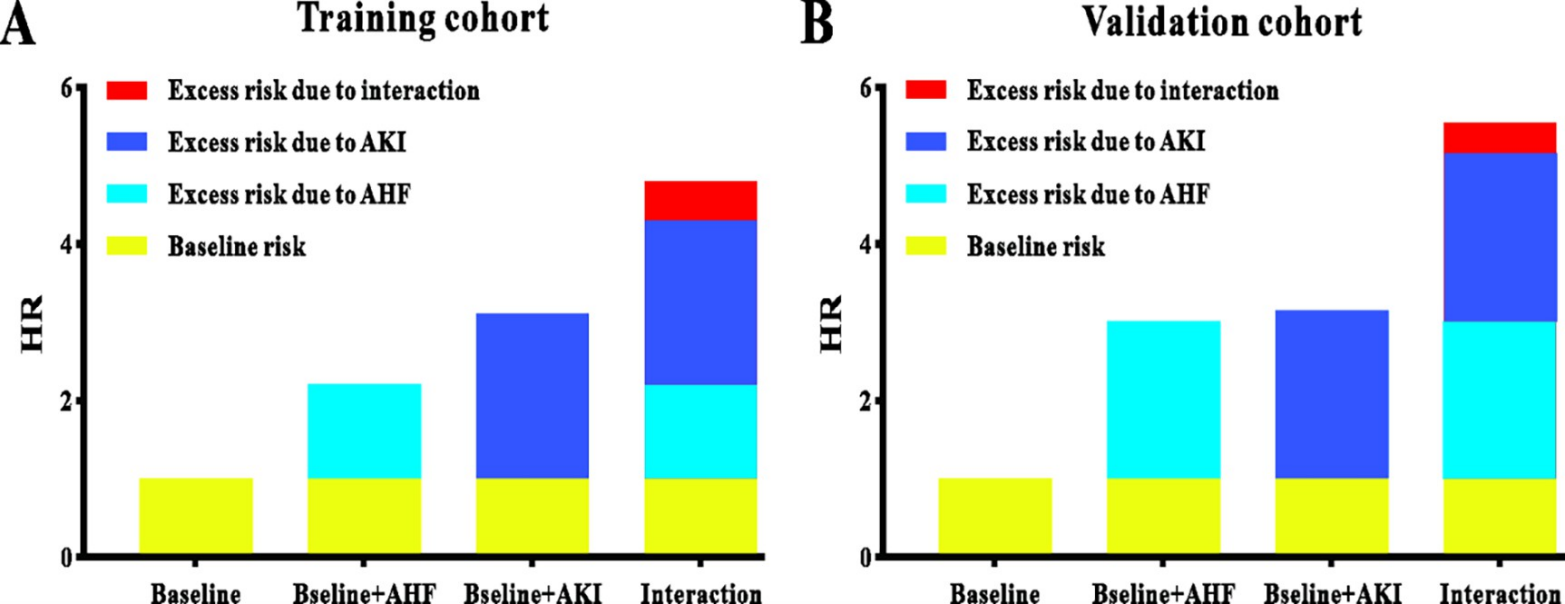
Relative risk with contributions from acute heart failure, acute kidney injury, or a combination of both (**A**) in the training cohort and (**B**) in the validation cohort.

Fig 4A and 4B displayed the extra risks owing to AHF, AKI, and their interaction in an analysis of in-hospital mortality adjusted for all risk variables, the risk of one complication (AKI or AHF), compared with no problems, was considerably higher. The risks of two complications surpassed the sum risk of AKI only and the AHF only both in the training cohort and in the validation cohort, showing a synergistic interacting impact of AHF and AKI.

## Discussion

In the current research, we aimed to elucidate the synergistic impact of AHF and AKI on in-hospital mortality of critically ill patients with sepsis. The findings indicated that a single problem was related with an increased risk of in-hospital mortality, the addition of a second difficulty exhibited substantial positive additive effects, giving evidence for interactions between these difficulties. The data used in this study were based on two large public databases with a total of 33,184 patients were finally included, including 20,626 patients in the training cohort extracted from the MIMIC-IV database and 12,558 patients in the validation cohort extracted from eICU-CRD database, this study included a large number of cases, and validated by another database, the results are solid. Moreover, to further remove possible influencing variables, subgroup analysis was undertaken which further validated the interaction effects of AKI and AHF on in-hospital mortality in critically ill patients with sepsis, demonstrated more solid findings in the current research.

The in-hospital mortality was common occurred in critically ill patients with sepsis. And the in-hospital mortality was 14.4% in the training cohort and 17.9% in the validation cohort, which was consistented with the result of Kong et al. (17.7%) which used the MIMIC-III database [21]. In recent years, many rseachers investigated the risk factors of in-hospital of mortality in patients with critically ill sepsis, in our study, age, vaopressors usage, SOFA score, OASIS score, APSIII score, GCS score, respiration rate, albumin, BUN and lactate were independently predictor of in-hospital of mortality in patients with critically ill sepsis both in the training cohort and in the validation cohort. Ren et al. previously reported that age, lactate, temperature, oxygenation index, BUN, lactate, Glasgow Coma Score (GCS), liver disease, cancer, organ transplantation, Troponin T(TnT), neutrophil-to-lymphocyte ratio (NLR), and CRRT, MV, and vasopress were risk factors of in-hospital mortality in ICU patients with sepsis based on MIMIC-III database [22]. Moreover, SOFA score, OASIS score, APSIII score are common score systems which used for prognosis prediction in the ICU setting. Previous study showed that SOFA, OASIS, SAPS II, LODS, and SAPS III scores could used for 28-day mortality prediction in sepsis patients [23]. In our study, SOFA score, OASIS score, APSIII score were identified as independently in-hospital mortality predictors of critically ill sepsis patients. Furthermore, our study also found albumin was a in-hospital mortality predictors of critically ill sepsis patients, which was similar to previous reported [24].

Previous studies indicated that both AKI and AHF are frequent consequences in critically ill individuals with sepsis [25,26]. One complication may raise therisk of death. However, the synergistic combination of two complications (AKI and AHF) on in-hospital mortality in critically unwell patients with sepsis remains mainly unexplained. Few studies studied the synergistic interplay of various problems in certain circumstances. Chen et al. [27] conducted a retrospective, observational cohort study to evaluate the joint effect of acute respiratory failure (ARF) and AKI on in-hospital mortality in patients with acute exacerbation of chronic obstructive pulmonary disease (AECOPD), the results demonstrated that AECOPD patients with ARF and AKI had 8.53 times, 8.99 times risk of in-hospital mortality, respectively, and those coexisted both ARF and AKI, its in-hospital mortality increased to 39.13 times. The relative excess risk owing to interaction was 22.62, the attributable percentage due to interaction was 0.59, and the synergy index was 2.46, demonstrating ARF and AKI had a strong synergic impact on in-hospital mortality. It may be assumed that an extra complication would synergistically increase to the chance of fatality. Whereas, it is not that the more issues happened in patients, the bigger the synergistic interaction effects would be identified. Kim et al. performed a retrospective, observational cohort study to investigate the synergistic interaction of AKI, acute respiratory failure and sepsis on short-term perioperative mortality in patients undergoing high-risk intraabdominal general surgery procedures, compared to those with no complication, the risk of short-term perioperative mortality for a single complication were 7.24 times, 10.8 times, and 14.2 times for sepsis, AKI, and acute respiratory failure, respectively. For 2 complications, the risks were 30.8 times, 42.6 times, and 65.2 times for acute respiratory failure coexisted with sepsis, AKI coexisted with sepsis, and acute respiratory failure coexisted with AKI, respectively. Finally, the risk for all three problems was 105 times. Positive additive interactions, suggesting synergism was discovered for each combination of two difficulties. However, the proportionate extra risk attributable to interaction for all three problems was not statistically significant [28]. In the current investigation, we also exhibited an additive positively synergistic interaction between AHF and AKI on in-hospital mortality in critically ill patients with sepsis both in the training group and in the validation cohort.

However, the actual processes driving the synergisms remained undiscovered. The proposed processes may owing to the interaction effect between kidney and heart in critically ill individuals with sepsis. Sepsis-induced cardiorenal syndrome (CRS) is one of the several organ dysfunctions reported in critically sick individuals with sepsis and CRS type 5 demonstrates concurrent cardiac and renal dysfunction which is typically related to sepsis [29]. In a sepsis state, elevated inflammatory markers and free radicals would produce endothelial calcification and dysfunction leading in poor perfusion of the heart and kidneys rendering them more prone to chronic renal and cardiac damage [30]. Moreover, macrovascular hemodynamic abnormalities in sepsis might lead to selective hypoperfusion of the renal medulla and consequently ischemic AKI and acute tubular necrosis [31]. Conversely, renal ischemia leads to elevated inflammatory mediators in the heart that result in expanded left ventricular dimensions and hence reduced systolic performance [32]. Worsening circulatory failure would also necessitate higher dosages of vasopressors and subsequently renal hypoperfusion and ischemia [33]. In addition, excessive intravenous fluids during resuscitation can cause visceral edema and abdominal or kidney intra-capsular compartment syndrome, with a further decline in the renal blood flow and fluid overload had a direct influence on the worsening of cardiac function by elevation of cardiac filling pressures [34]. Hence, the synergistic combination of AKI and AHF may be owing to the crosstalk between kidney and heart in sepsis-induced CRS. And the particular processes are required to be researched in the future.

Several limitations should be highlighted in this investigation. First, the current research was a retrospective analysis based on two public databases, and the findings should be further validated by future prospective studies or randomized controlled trials. Furthermore, BNP values were important for the diagnosis of AHF patients, however, BNP values were not obtained for more than half of the patients in this study (89.1% in the training cohort and 70.3% in the validation cohort missing), this will cause an unrobust result to this study. Meanwhile, multi-organ failure may be detected in about 40–60% of all patients presenting with sepsis, including renal and cardiac dysfunction [35]. Except for the heart and kidney, other organs including the liver and lung may increase the risk of in-hospital mortality, and this research exclusively focuses on the heart and kidney.

## Conclusions

Our results indicated that AKI and AHF may independently enhance the risk of in-hospital mortality in critically unwell patients with sepsis. And individuals experienced two problems that surpass the total risk of each complication, showing a synergistic combination of AHF and AKI, and this impact may related to the crosstalk between heart and kidney. This research offered doctors a suggestion that when critically ill patients with sepsis coexisted with AKI and AHF, it would create far-reaching risks of death than those with one condition.

## Supporting information

S1 Checklist (DOCX)

S1 Table The baseline characteristics of all patients in validation cohort categorized by AHF and AKI. (DOCX)

S2 Table Cox analyses of factors associated with in-hospital mortality in validation cohort. (DOCX)
