# Supplementary material for: Interaction of acute heart failure and acute kidney injury on in-hospital mortality of critically ill patients with sepsis: A retrospective observational study
Source: PLoS One. 2023 Mar 8;18(3):e0282842. doi: 10.1371/journal.pone.0282842 (PMC9994701; doi:10.1371/journal.pone.0282842)
Supplement: S2 Table — (DOCX) [file pone.0282842.s003.docx]

**S2 Table. Cox analyses of factors associated with in-hospital mortality in validation cohort**

|  | Univariate analysis | | Multivariate analysis | |
| --- | --- | --- | --- | --- |
|  | HR (95%CI) | P value | HR (95%CI) | P value |
| Age | 1.03 (1.02-1.03) | <0.001 | 1.02 (1.01-1.02) | <0.001 |
| Gender, male | 1.01 (0.93-1.10) | 0.730 |  |  |
| Ethnicity |  |  |  |  |
| White | 1.05 (0.92-1.20) | 0.447 |  |  |
| Black | 0.89 (0.75-1.06) | 0.191 |  |  |
| Others | Ref. | - |  |  |
| Body mass index | 1.00 (0.99-1.00) | 0.115 |  |  |
| Comorbidities |  |  |  |  |
| Hypertension | 1.03 (0.95-1.12) | 0.441 |  |  |
| Diabetes | 0.95 (0.87-1.04) | 0.299 |  |  |
| Congestive heart failure | 1.21 (1.09-1.35) | <0.001 | 0.98 (0.87-1.10) | 0.729 |
| Myocardial infarct | 1.11 (0.95-1.30) | 0.172 |  |  |
| Chronic kidney disease | 1.22 (1.10-1.36) | <0.001 | 1.16 (1.02-1.43) | 0.021 |
| Liver disease | 1.12 (0.97-1.33) | 0.217 |  |  |
| COPD | 1.20 (0.90-1.44) | 0.329 |  |  |
| Atrial fibrillation | 1.27 (1.13-1.42) | <0.001 | 0.92 (0.62-1.38) | 0.693 |
| Valve disease | 1.37 (1.13-1.67) | <0.001 | 1.15 (0.94-1.41) | 0.170 |
| Cardiac arrhythmia | 1.28 (1.14-1.43) | <0.001 | 1.09 (0.74-1.61) | 0.676 |
| PVD | 1.25 (1.06-1.49) | 0.010 | 0.96 (0.80-1.14) | 0.636 |
| Hypothyroidism | 1.08 (0.97-1.20) | 0.146 |  |  |
| Charlson index | 1.14 (1.13-1.16) | <0.001 | 1.12 (1.10-1.14) | <0.001 |
| Interventions |  |  |  |  |
| MV use | 0.68 (0.63-0.75) | <0.001 | 0.88 (0.80-0.98) | 0.022 |
| RRT use | 0.85 (0.75-0.95) | 0.019 | 0.79 (0.68-0.91) | 0.001 |
| Vasopressors | 0.51 (0.47-0.56) | <0.001 | 0.79 (0.71-0.86) | <0.001 |
| Drug usage |  |  |  |  |
| ACEI/ARB | 0.91 (0.83-1.02) | 0.109 |  |  |
| β blockers | 0.79 (0.73-0.87) | <0.001 | 0.81 (0.74-0.89) | <0.001 |
| CCB | 1.00 (0.88-1.13) | 0.956 |  |  |
| Diuretic | 0.83 (0.76-0.90) | <0.001 | 0.75 (0.69-0.83) | <0.001 |
| Severity scores |  |  |  |  |
| SOFA | 1.14 (1.12-1.15) | <0.001 | 1.06 (1.04-1.08) | <0.001 |
| OASIS | 1.05 (1.04-1.06) | <0.001 | 1.02 (1.01-1.03) | <0.001 |
| APS III | 1.02 (1.01-1.03) | <0.001 | 1.03 (1.01-1.04) | <0001 |
| GCS | 0.94 (0.93-0.95) | <0.001 | 0.97 (0.96-0.98) | <0.001 |
| Vital signs |  |  |  |  |
| Heart rate | 1.00 (0.99-1.00) | 0.479 |  |  |
| Respiratory rate | 1.02 (1.01-1.03) | <0.001 | 1.01 (1.00-1.02) | 0.008 |
| MAP | 0.99 (0.99-1.01) | 0.172 |  |  |
| Laboratory results |  |  |  |  |
| WBC | 1.00 (0.99-1.01) | 0.156 |  |  |
| HGB | 0.95 (0.94-0.97) | <0.001 | 0.99 (0.92-1.01) | 0.989 |
| Platelets | 1.00 (0.99-1.01) | 0.147 |  |  |
| Albumin | 0.67 (0.63-0.71) | <0.001 | 0.75 (0.70-0.80) | <0.001 |
| Bilirubin | 1.02 (0.98-1.05) | 0.574 |  |  |
| Aniongap | 1.03 (1.02-1.03) | <0.001 | 1.01 (0.99-1.02) | 0.223 |
| Bicarbonate | 0.98 (0.95-1.01) | 0.104 |  |  |
| BUN | 1.01 (1.01-1.02) | <0.001 | 1.01 (1.00-1.04) | 0.034 |
| Creatinine | 1.02 (0.99-1.03) | 0.219 |  |  |
| Lactate | 1.16 (1.14-1.17) | <0.001 | 1.11 (1.10-1.13) | <0.001 |
| BNP (tested) | 0.93 (0.86-1.02) | 0.127 |  |  |
| Complications |  |  |  |  |
| Neither AHF nor AKI | Ref. | - | Ref. | - |
| AHF only | 2.07 (1.76-2.43) | <0.001 | 2.01 (1.69-2.38) | <0.001 |
| AKI only | 2.60 (2.36-2.87) | <0.001 | 2.15 (1.94-2.38) | <0.001 |
| Both AHF and AKI | 5.44 (4.76-6.22) | <0.001 | 4.54 (3.92-5.25) | <0.001 |

HR, hazard ratio, 95%CI, 95% confidence index, COPD, chronic obstructive pulmonary disease, PVD, peripheral vascular diseases, MV, mechanical ventilation, RRT, renal replacement therapy, ACEI/ARB, Angiotensin converting enzyme inhibitors/Angiotensin receptor blockers, CCB, Calcium calcium blockers, SOFA, sequential organ failure assessment, OASIS, oxford acute severity of illness score, APSIII, acute physiology score III, GCS, glasgow coma scale, MAP, mean arterial pressure, WBC, white blood cell, BUN, blood urea nitrogen, BNP, brain natriuretic peptide, AHF, acute heart failure, AKI, acute kidney injury.
